# Infrequent mutation of the tumour-suppressor gene Smad4 in early-stage colorectal cancer

**DOI:** 10.1038/sj.bjc.6600733

**Published:** 2003-02-10

**Authors:** C Mamot, G Mild, J Reuter, U Laffer, U Metzger, L Terracciano, J-L Boulay, R Herrmann, C Rochlitz

**Affiliations:** 1 Division of Oncology, University Hospital Basel, 4031 Basel, Switzerland; 2 Department of Research, University Hospital Basel, 4031 Basel, Switzerland; 3 The Swiss Group for Clinical Cancer Research (SAKK), 3000 Bern, Switzerland; 4 Institute of Pathology, University Hospital Basel, 4031 Basel, Switzerland

**Keywords:** DPC4, loss of heterozygosity (LOH), Smad4, TGF*β*, tumorigenesis

## Abstract

Smad4 is a candidate tumour-suppressor gene identified recently on chromosome 18q21.1. Both alleles are inactivated in nearly one-half of pancreatic carcinomas, but its role in the tumorigenesis of other tumours is still unknown. The aim of this study was to investigate the potential involvement of the Smad4 locus in early-stage colorectal cancers (stages I–III) in tumour samples from a randomised multicentre trial. Of a large collection of DNA samples, 73 with a loss of one allele of the Smad4 gene were analysed for the presence of point mutations in the remaining gene. Patients, from whom biopsies were isolated, were part of a previous randomised multicentre study of the Swiss Group for Clinical Cancer Research on the benefit of adjuvant chemotherapy (SAKK study 40/81). Mutation analysis was restricted to the highly conserved C-terminal domain (exons 8, 9, 10 and 11) of Smad4, using PCR and single-strand conformational variant analysis. Two of the 73 patients (3%) with loss of one allele of Smad4 had a point mutation in the remaining allele. These results indicate that whereas Smad4 point mautations are prevalent in pancreatic carcinoma, they are infrequent in early stages (I–III) of colorectal cancer.

Deletion of a chromosomal region is a frequent cytogenetic alteration observed in carcinogenesis. The loss of tumour-suppressor genes has been reported in numerous types of human tumours, in particular those of the gastrointestinal tract (Vogelstein *et al*, 1988). APC and p53 have been widely recognised as important tumour-suppressor genes inactivated during colorectal carcinogenesis. Several other tumour-suppressor genes have been located on chromosomes 1p, 8p, 18q and 22q. In particular, loss of heterozygosity (LOH) at 18q21 is correlated with carcinomas of the colon, and other tumours such as pancreatic carcinoma, renal cell carcinoma, melanoma and breast carcinoma (Schutte *et al*, 1996; Barbera *et al*, 2000). Much of the interest in this region arose because reports indicated that 18q losses are associated with high metastatic potential and reduced patient survival (Iino *et al*, 1994).

In fact, several potentially cancer-related genes map to the 18q21 region, including bcl-2, gastrin-releasing peptide gene and cellular homologue of yes-1. However, none of these have been observed to be mutated in colorectal cancer (CRC). Fearon *et al*. (1990) identified another tumour-suppressor gene localised on 18q21, designated DCC for deleted in colorectal cancer. However, there have been several cases in which loss of expression did not correlate with LOH (Kikuchi-Yanoshita *et al*, 1992), and mutation in the coding region of the DCC gene has been infrequently detected (Cho *et al*, 1994; Sato *et al*, 2001). Owing to the controversial evidence as to the role of DCC in cancer, additional genetic analysis of the 18q21 region led to the identification of other potential tumour-suppressor genes, including three candidate tumour-suppressor genes: Smad2, Smad4 and Smad7. These genes are involved in signal transduction of the TGF*β* signalling pathway. Members of the transforming growth factor (TGF)-*β* family transmit their signals from the plasma membrane to the nucleus through combinations of serine/threonine kinase receptors and their downstream effectors, known as Smads. After the Smad4 (MADH4) gene was isolated from the same region as a tumour-suppressor gene for pancreatic cancer (Hahn *et al*, 1996), mutation analysis of this gene has been carried out in various cancers. In recent studies, Smad4 was identified as a genetic target in pancreatic carcinomas, inactivated through homozygous deletion (*n*=5), intragenic mutation (*n*=3) and lack of protein (*n*=2) in 10 out of 16 pancreatic cell lines (Barbera *et al*, 2000). Furthermore, it could be shown that when genetically inactivated this tumour-suppressor in the TGF*β* signalling pathway represents a prognostic factor in invasive pancreatic cancer influenced by Smad4 status (Tascilar *et al*, 2001). In addition to observations in pancreatic carcinomas, Smad4 is also known as a gene involved in juvenile polyposis tumour predisposition syndrome (Howe *et al*, 1998; Huang *et al*, 2000). Mutations of the Smad4 gene have been detected in some colorectal cancers, but its role in this specific cancer remains unclear. The frequencies of mutations (5–45%) have been found to be low (Takagi *et al*, 1996; MacGrogan *et al*, 1997; Ohtaki *et al*, 2001), but data originated from relatively small studies, and the tumour populations examined were inhomogeneous explaining the broad range of incidences found.

The aim of this study was to further expand these data by Smad4 mutation analysis of a large set of early-stage (I–III) colorectal cancer patients treated in a randomised multicentre trial of 5-fluorouracil (5-FU)/Mitomycin C adjuvant chemotherapy of the Swiss Group for Clinical Cancer Research (SAKK study 40/81). Owing to the significance of LOH in colorectal cancer and the role of the remaining gene, this study was focused on patients with an allelic loss of one Smad4.

## METHODS

### Patients

Patients from whom biopsies were isolated, were part of a previous randomised multicentre study of the SAKK on the benefit of treatment with adjuvant chemotherapy between 1981 and 1987 (Laffer, 1995). Deoxyribo nucleic acid (DNA) samples of these patients were extracted from tumour as well as from healthy tissue derived from the same patient in order to perform genetic analyses. Paraffin-embedded material was available from 329 of the 505 patients. To investigate genetic alterations in the 18q21 region in these tumours, a gene dosage study of the tumour-suppressor genes Smad2, Smad4 and DCC was performed (Boulay *et al*, 1999). For technical reasons, high-quality DNA for analysis was available from 294 patients only. Individual dosage of the Smad4 gene showed a total deletion frequency (one or both alleles) of 68% when compared to normal tissue. In total, 167 patients (=57%) were detected with an allelic loss of one Smad4 copy. In this study, we randomly chose 73 of these 167 patients to search for the presence of point mutations in the remaining gene. After analysis of these 73 out of 167 patients, two point mutations of Smad4 had been detected, and for statistical reasons, further mutation analysis in the remaining 94 out of 167 patients did not seem necessary to substantiate our finding.

### Gene copy status scoring

Genomic samples from 294 patients were tested for copy dosage of the Smad4 gene using TaqMan quantitative real-time PCR (Perkin-Elmer, Huenenberg, Switzerland). Copy status of the Smad4 gene was determined by comparing tumour DNA to DNA from normal tissue derived from the same patient as described previously (Boulay *et al*, 1999).

### Duplex PCR

Polymerase chain reaction (PCR) amplification on DNA was performed in 15 *μ*l reaction volume, containing 1.5 *μ*l 10×PCR buffer (Perkin-Elmer, Huenenberg, Switzerland), 10 mM 2′desoxyribonucleosoid-5′-triphosphate (dNTPs), 20 *μ*M of each primer, 1 U of AmpliTaq Gold (Perkin-Elmer, Huenenberg, Switzerland), ^32^P Oligo (2.5 *μ*l 10×Buffer, 20 *μ*M forward primer, 1 *μ*l PNK and 1 *μ*l *γ*^32^P-adenosine triphosphate (ATP) incubated 30 min at 37°C) and 100 ng DNA. Duplex PCR for Smad4 gene was done using primers EX 8/1 and EX 8/2, EX 9/1 and EX 9/2, EX 10/1 and EX 10/2, and finally EX 11/1 and EX 11/2 (Table 1

**Table 1:** Primer–sequences designed for duplex-PCR

**Primer**	**Forward**	**Reverse**
EX 8/1	5′-GAAAGCCTTATATC TTTCTC-3′	5′CACGTATCCATCAACAGTAA-3′
EX 8/2	5′-TCCTTCAAGCTGCCCTATTG-3′	5′-CAATTTTTTAAAGTAACTATCTGA-3′
EX 9/1	5′-TATTAAGCATGCTATACAATCTG-3′	5′-GTGGTCACTAAGGCACCTGA-3′
EX 9/2	5′-TAAAGGTGAAGGTGATGTTT-3′	5′-CAAATAGAGCTTTAAGTCTA-3′
EX 10/1	5′-GTCAGGCATTGGTTTTTAATG-3′	5′-ATCCTGGGCCAGGGATGTTT-3′
EX 10/2	5′-AAACATCCCTGGCCCAGGAT-3′	5′-CAAAAATGTCATCATCCC AGT-3′
EX 11/1	5′-AAGAGATCACCCTGTCCCTCT-3′	5′-CCAGCAAGGTGTTTCTTTGA-3′
EX 11/2	5′-GGATTACCCAAGACAGAGCA-3′	5′-GTATTTTGTAGTCCACC ATC-3′

). Polymerase chain reaction conditions were as follows: 40 amplification cycles of denaturation at 94°C for 45 s, annealing at 55°C for 60 s, and extension at 72°C for 60 s, followed by one cycle at 72°C for 10 min. Amplification products were loaded on a 0.4 mm acrylamide gel in a ‘Model S2 Sequencing Gel Electrophoresis Apparatus (Life Technologies, Switzerland)’. Electrophoresis settings are: 1800 V, 35–40 mA, 60 VA and 120 min. Polymerase chain reactions without DNA templates were performed as negative controls. Bands were subsequently cut out from the single-strand conformation polymorphism (SSCP)-gel and reamplified in a PCR.

### Sequencing analysis

Reamplification of DNA was performed in a 50 *μ*l reaction volume, containing 5 *μ*l 10×PCR buffer (Perkin-Elmer, Huenenberg, Switzerland), 10 mM dNTPs, 20 *μ*M forward and backward primer, 1 U of AmpliTaq Gold (Perkin-Elmer, Huenenberg, Switzerland) and 38 *μ*l H_2_O. PCR conditions were as follows: 35 amplification cycles of denaturation at 94°C for 45 s, annealing at 55°C for 60 s and extension at 72°C for 60 s, followed by one cycle at 72°C for 10 min. Sequencing analysis was performed by Microsynth (Basel, Switzerland) on a fluorescence-based DNA sequencer that utilises capillary electrophoresis with 96 capillaries operating in parallel.

## RESULTS

**Figure 1:**
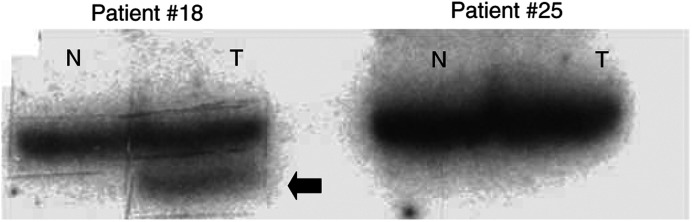
SSCP analysis of Smad4 in early-stage colorectal cancer. Representative results of PCR–SSCP analysis using EX9/2f and EX9/2r primers (exon 9). Patient No. 18 shows a migration alteration in tumour DNA (T) compared to normal tissue DNA (N) defined as an SSCP, whereas patient No. 25 does not display any polymorphism. Extra bands (
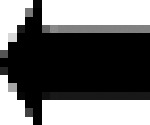
) were subsequently cut out from the SSCP-gel and reamplified in a PCR before subjected to sequencing analysis.

Among the 294 tumours for which gene dosage data (Smad2, Smad4 and DCC) were available, 167 tumours (57%) showed heterozygous loss of Smad4 (Boulay *et al*, 1999), and 73 out of 167 samples were randomly chosen for mutation analysis. Of these, only two (3%) carried point mutations in Smad4 in tumour but not the corresponding healthy tissue, as demonstrated by PCR–SSCP (Figure 1

). The two mutations were located in the highly conserved C-terminal Smad4 homology region. One confirmed point mutation was found in exon 9 and another point mutation in exon 11. Both mutations were confirmed by direct sequencing analysis showing one mutation resulting in an amino-acid change from arginine to serine; the second mutation led to an exchange of alanine to valine (Table 2

**Table 2:** Mutations of Smad4 gene detected in colorectal cancer

**Patient**	**Exon**	**Alteration**	**Effect**
18	9	AGA→AGC	Arginine (R)→Serine (S)
376	11	GCT→GTT	Alanine (A)→Valine (V)

Two out of 73 patients (3%) with point mutations of Smad4.

).

However, a caveat in the interpretation of our data needs to be mentioned: informative and reproducible data were available from a total of 174 complete exons (8, 9, 10 and 11) derived from the 73 defined patients. This shortcoming of our data was because of technical problems in the analysis, caused by the sometimes poor quality of the DNA, as is often observed with nucleic acids isolated from paraffin-embedded tissue. Nevertheless, since the exons for which interpretable results were available were equally distributed between the eight different amplicons we used in our study, our conclusion of a very low frequency of Smad4 point mutations in the population studied is not put into question by this technical shortcoming.

## DISCUSSION

Smad proteins are a novel family of proteins that function downstream of serine/threonine kinase receptors to transduce signals for members of the TGF*β* superfamily (Massague, 1996). The three Smads (Smad2, Smad4 and Smad7) encoded in the 18q21 chromosomal region participate in the signalling mechanisms subsequent to TGF*β*-receptor complex formation. Smad4, a co-Smad of Smad2, is known as a tumour-suppressor gene in different cancer types. Tumour-suppressor genes are often inactivated when one allele acquires a mutation and the second allele is lost, typically through deletion (Cavenee *et al*, 1983). The tumour-suppressor gene p53 represents just one example for this classic concept (Miller *et al*, 1992), while for another tumour-suppressor gene, DCC, these findings could not be confirmed (Sato *et al*, 2001). Our screen of 73 patients with early-stage colorectal cancer (I–III) carrying a loss of one Smad4 allele identified two mutations of the remaining allele (3%), a finding that is in accordance with results described in the literature (Schutte *et al*, 1996; Miyaki *et al*, 1999).

Mutation analysis was restricted to exons 8, 9, 10 and 11 of Smad4, which together span the entire conserved C-terminal Smad4 homology region. Since 90% of the Smad4 mutations reported are located in that highly conserved region, the number of undetected mutations is expected to be low when the analysis is restricted to these mutation hot spots (Hahn *et al*, 1996; Takagi *et al*, 1996; Kong *et al*, 1997). The low rate of point mutations detected by our method deserves further comments: SSCP has been shown to be a highly sensitive method to identify mutations in PCR-generated fragments. The sensitivity of SSCP analysis is widely disputed in the literature, with reports ranging from 35% (Sarkar *et al*, 1992) to nearly 100% (Orita *et al*, 1989). Of course, certain mutations may not be detected using this method. Furthermore, it is possible that some of our tumours had large intragenetic deletions of Smad4, which would have been missed with the detection method used. However, the single factor having the greatest effect on SSCP sensitivity is the size of the DNA fragments. An optimal size of 200 base pairs (bp) or less was used in our study (160–180 bp), which is described as the most sensitive for single-base substitutions (Sheffield *et al*, 1993).

Among the mediators of TGF*β* signalling encoded by the 18q21 chromosomal region, two were identified as involved in activating TGF*β* signalling: Smad2 and Smad4, and one, in the inhibition of TGF*β* signalling: Smad7. Thus, one could have expected that inactivation of Smad4 might result in a TGF*β* resistance that would favour tumour expansion. Interestingly, the patients with deletion of Smad4 did not show a significantly worse prognosis than those without a deletion (Boulay *et al*, 2002). In contrast, in the same population, Smad4 seemed to be a predictive marker for 5FU/mitomycin adjuvant chemotherapy. However, whether Smad4 plays a key role in tumorigenesis of colorectal cancer is still unclear.

To date, a significant number of Smad4 point mutations have been found only in pancreatic carcinomas (50–60%), biliary tract carcinomas (15%) or colorectal carcinomas (5–20%) (Hahn *et al*, 1996; Schutte *et al*, 1996; MacGrogan *et al*, 1997). Although the existence of additional unknown target tumour-suppressor genes in the region of 18q21 cannot be ruled out, recently published results strongly suggest a significant contribution of Smad4 gene inactivation in advanced tumour stages. Metastatic colorectal carcinomas including carcinomas metastasised to the liver showed a considerably higher frequency (31–35%) than invasive carcinomas without distant metastasis (7%) (Miyaki *et al*, 1999; Ohtaki *et al*, 2001). Our findings of less than 5% point mutations are at the lower end of the spectrum and confirm the low frequency of point mutations of Smad4 in early-stage colorectal cancer without distant metastasis. The limitation to patients with loss of one Smad4 allele–initially used to select a population with a presumably high mutation frequency–is one possible theoretical explanation for our findings. However, in pancreatic and biliary tract carcinomas, patients with LOH represent a group with an especially high point mutation frequency in the remaining gene, making this explanation highly unlikely (Hahn *et al*, 1998; Barbera *et al*, 2000).

Other possible explanations for the absence of Smad4 point mutations in colorectal cancer at this stage include methylation changes at the promoter and alternative splicing or changes in mRNA stability (Roth *et al*, 2000). The importance of genes that undergo alterations at low prevalence, however, may as yet be underestimated. Such events may contribute significantly to the genetic variety within a tumour type and, thus, to the complexity of human tumorigenesis. Since it is likely that many alterations of low prevalence exist in human cancers, an individual tumour might still acquire several of these different alterations with a high probability, making low prevalence alterations a powerful driving force of the carcinogenic process.

In conclusion, our findings indicate that Smad4 point mutations are infrequent in early stages of colorectal cancer. However, it cannot be completely ruled out that inactivation of Smad4 could be a common genetic event at later stages of colorectal cancer. Future research comparing early and advanced stages is required to investigate the tumour-suppressor pathway in colorectal cancer and to redefine the role Smad4 signalling plays in tumorigenesis.
